# *Pseudaptinus (Thalpius) nobilis* Liebke, new to the United States, and a key to the species of subgenus *Thalpius* LeConte in North America, including Mexico (Coleoptera, Carabidae, Zuphiini)

**DOI:** 10.3897/zookeys.147.1918

**Published:** 2011-11-16

**Authors:** Peter W. Messer

**Affiliations:** 1 4315 W. Riverlake Dr., Mequon, WI 53092, USA

**Keywords:** Coleoptera, Carabidae, Zuphiini, *Pseudaptinus*, *Thalpius*, North America

## Abstract

The Mexican carabid species *Pseudaptinus (Thalpius) nobilis* Liebke is documented from the United States for the first time based on two specimens captured in southeastern Texas. A new taxonomic key distinguishes the 10 members of subgenus *Thalpius* known in North America, including Mexico. *Pseudaptinus (Thalpius) dorsalis*, which is found to be highly variable in its dorsal coloration, is compared to similar *Pseudaptinus (Thalpius) hoegei*. Geographic ranges are extended for several species.

## Introduction

*Thalpius* LeContein Carabidae: Zuphiiniis currently treated as a subgenus of *Pseudaptinus* Laporte (Baehr 1985, Lorenz 2005). The subgenus is a Neotropical-Australian group that consists of 33 species (11 Australian, 22 New World; Robert Davidson *in litteris,* corrections to Lorenz (2005) in which several species are placed in the wrong subgenus and several synonyms are incorrectly listed as species), of which eight were reported by Bousquet and Larochelle (1993) to occur in the United States. Liebke (1934) described *Pseudaptinus (Thalpius) nobilis* with the type locality given simply as “Mexico”. Reported here is the first capture of *Pseudaptinus (Thalpius) nobilis* in the United States, specifically in southeastern Texas, which brings the total count to nine species. *Pseudaptinus (Thalpius) nobilis* is easily separated from other adults of the subgenus by the diagnostic combination of elongated middle antennomeres, markedly reduced eyes, and a black head contrasting with a dark red-brown body. It is further distinguished in a newly synthesized taxonomic key to the 10 species of subgenus *Thalpius* known in North America, including Mexico. The key is a consolidation of information that I obtained from specimen studies and a literature review of Bousquet and Larochelle (1993), LaBonte (1996), LeConte (1879), Liebke (1934), Darlington (1934), and Van Dyke (1926). *Pseudaptinus (Thalpius) dorsalis*, which is found to be highly variable in its dorsal coloration, is compared to similar *Pseudaptinus (Thalpius) hoegei*. Geographic ranges are extended for several species.

## Material

Using the key and original description by Liebke (1934), I determined two adult beetle specimenscaptured in southeastern Texas to be *Pseudaptinus (Thalpius) nobilis*. They compared well to typical examples of the same species about 800 miles to the south in the state of Veracruz, Mexico (36 miles south of Acayucan, 5-VII-1971, at light). The two specimens were pinned females bearing the same label data: TX, Live Oak County, Calliham, Choke Canyon State Park, 24-June-2009, black light trap, B. Buchli. A prior scientific collection permit had been obtained by the private collector (personal communication). Choke Canyon State Park, despite its name, has no canyon. The local terrain is characterized as a semi-arid, gently-rolling brushland with soils ranging from loamy sand to heavy clay. The area includes lakes, streams, and dense thickets of mesquite and blackbush acacia.

## Discussion

Other than associations with moist soil and occurrences under stone cover during the day, almost nothing else is known about the natural history of subgenus *Thalpius*. Flying adults are usually collected at nocturnal light sources as was the case in this report. The 10 species of subgenus *Thalpius* that are currently known to occur in North America (including Mexico) are fully cited here:

*Pseudaptinus (Thalpius) cubanus* (Chaudoir, 1877); *Pseudaptinus (Thalpius) deceptor* Darlington, 1934; *Pseudaptinus (Thalpius) dorsalis* (Brullé, 1834); *Pseudaptinus (Thalpius) hoegei* (Bates, 1883); *Pseudaptinus (Thalpius) horni* (Chaudoir, 1872); *Pseudaptinus (Thalpius) microcephalus* (Van Dyke, 1926); *Pseudaptinus (Thalpius) nobilis* Liebke, 1934; *Pseudaptinus (Thalpius) pygmaeus* (Dejean, 1826); *Pseudaptinus (Thalpius) rufulus* (LeConte, 1851); *Pseudaptinus (Thalpius) simplex* Liebke, 1934.

All species are now known to inhabit the contiguous United States except for *seudaptinus simplex* which is limited to Mexico. None occurs in Canada. The geographic range of subgenus *Thalpius* in the United States follows a somewhat U-shaped pattern from Pennsylvania southward to Florida, across the entire southern one-third of the country to California, and northward to Oregon. The northern limit in Oregon was reported for *seudaptinus rufulus* by LaBonte (1996).

Previously *seudaptinus nobilis* had been known only from Mexico. It is distinguished from other species in the taxonomic key presented here. The collection site in southeastern Texas reported here is about 800 miles north of the Veracruz site in Mexico which yielded examples of the same species. Both sites are within 70 miles of the Gulf of Mexico. It is reasonable to assume that the Gulf Coastal Plain in Mexico, east of the parallel Sierra Madre Oriental, provides a narrow flat corridor for the northward migration of many insect species. I am left wondering if the simultaneous capture of two female specimens of *seudaptinus nobilis* in southeastern Texasrepresentsmerelyan “accidental” occurrence or whether it signifies an established breeding population in the United States. More field work and surveys of collection material are needed.

I observed marked color variation in large series of specimens that otherwise fit *Pseudaptinus (Thalpius) dorsalis* (Brullé), a species found throughout southeastern United States and Mexico. The literature typifies the coloration as ferruginous with a contrasting dark cloud on the posterior-medial aspect of the elytra. However, most observed specimens (in the range 70-90%) were entirely cloudless and they varied in color from uniform rufotestaceous individuals to some with darker brownish forebody that contrasted with lighter elytra. I saw both clouded and cloudless *Pseudaptinus dorsalis* in a color continuum from the same collection event. The present key takes both of these forms to branches occupied by similar *Pseudaptinus (Thalpius) hoegei* (Bates), primarily a Mexican species with likely infrequent occurrences into southern Texas. The available literature provided neither an adequate description of *Pseudaptinus hoegei* nor did it offer comparisons with *Pseudaptinus dorsalis*. Instructive online images under the type name *Diaphorus högei* Bates, 1883 are available in the MCZ Type Database at Harvard College. These images show the dorsum uniformly brownish without an obvious dark sutural cloud on elytra. However, I confirmed clouded elytra in several Mexican specimens designated as *Pseudaptinus hoegei* in the Smithsonian Institution. *Pseudaptinus horni*, normally with clouded elytra, is known to be occassionally cloudless. The taxonomic key presented here takes into account the variation in elytral color patterns (clouded vs cloudless) as manifested by *Pseudaptinus horni*, *Pseudaptinus dorsalis*, and *Pseudaptinus hoegei*. The latter two species are ultimately distinguished on the basis of eye and temple proportions. Additional study of *Pseudaptinus dorsalis* and *Pseudaptinus hoegei* is needed to better understand their morphologic and geographic differences.

### Key to the adult species of *Pseudaptinus (Thalpius)* in North America, including Mexico

Previously published (incomplete) keys relied heavily on dorsal color and on the following comparative measurements. Abbreviation L/W is the ratio of length to width of the middle antenna segments (5^th^ or 6^th^ antennomeres). Care is taken to view the broadest aspect of each segment. The ratio E/T is the longitudinal diameter of eye to the length of the posterior temple before the neck constriction, best observed from above. The characteristic values assigned to these ratios are approximations which are usually appreciated visually without exact measurements. Minor adjustments of the published values were made in some cases. Apparent body length, given in millimeters, is measured from mandible tip to abdominal apex. The known Western Hemisphere range is given for each species. New regional extensions that I observed are superscribed **^1^**. Those superscribed **^2^** are additional records confirmed and provided by Robert L. Davidson (Carnegie Museum of Natural History, PA).

**Table:** 

1	Dorsum distinctly dull, uniformly dark pinkish brown, finely and very closely punctate; 5.5-6 mm. (FL; Bahamas, Caymans, Dominican Republic^2^, Cuba)	*Pseudaptinus cubanus*
–	Dorsum shiny, rufotestaceous to plain dark brown, more coarsely punctate in most species	2
2	Antennomeres 4-10 squarish, as long as wide (L/W = 1)	3
–	Antennomeres 4-10 distinctly elongated (L/W > 1)	4
3	Elytral striae rather irregularly but seriately punctate except apically, the punctures much coarser than those of the intervals; pronotum narrower and basal angles more minutely prominent; dorsum dark (reddish to blackish brown) usually with head and elytral disc darkest; eye prominent (E/T = 2); mostly 4.2-5 mm (southeastern US quadrant: AL, AR, FL, GA, LA, MS, NC^2^, eastern OK^ 1, 2^, SC, TN^2^, eastern TX^1^, VA^2^; Cuba)	*Pseudaptinus pygmaeus*
–	Elytral striae not evidently seriately punctate, not more coarsely punctate than intervals; pronotum broader, basal angles more broadly prominent; uniformly reddish to dark brown; 5-6 mm. (FL, southeastern TX^1^; Caymans, Cuba, Dominican Republic^2^)	*Pseudaptinus deceptor*
4	Body length less than 5 mm; dorsum uniformly rufotestaceous; head small, distinctly narrower than pronotum; eyes rather small and flattish; elytra with striae poorly defined, intervals flat; location in southern California; 4.75 mm. (CA)	*Pseudaptinus microcephalus*
–	Body length usually at least 5 mm; combination not as above	5
5	Elytra with posterior sutural dark cloud; middle antennomere ratio L/W = 1.75-2	6
–	Elytra without dark cloud; middle antennomere ratio L/W various	8
6	Body length ≥ 6 mm; elytral intervals flat to slightly convex, densely punctate; E/T = 1.5 with temples gently curved; middle antennomeres with L/W = 2; dark sutural cloud distinct against rufotestaceous dorsum; 6-7 mm.(southwestern US quadrant: AZ, CA, NM, NV, OK^2^, TX; Mexico^1^)	*Pseudaptinus horni*
–	Body length ≤ 6 mm; elytral intervals distinctly convex, sparsely and finely punctate; middle antennomeres with L/W = 1.75; sutural cloud often less distinct	7
7	Body length usually ≤ 5.5 mm; eyes larger; temples strongly curved, shorter than in *Pseudaptinus horni* (E/T = 1.5-1.9); sutural cloud more often faint or absent; mostly ferruginous with forebody sometimes darker; 4.7-5.5 mm. (southeastern US quadrant: AL^2^, AR, DC, FL, GA^2^, LA, OK^2^, PA^2^, SC, TX; Bahamas, Cuba, Dominican Republic, Jamaica, Mexico^1^ )	*Pseudaptinus dorsalis*
–	Body length 5.6-6 mm; eyes smaller; temples gently curved, longer against small eyes as compared to *Pseudaptinus horni* (E/T = 1-1.5); more uniformly brownish; (south-central US: MS*,* TX; Mexico)	*Pseudaptinus hoegei*
8	Body length ≥ 6 mm; middle antennomere ratio L/W = 2; see also key step #6	*Pseudaptinus horni*, an occasional specimen which lacks the sutural dark cloud on elytra. This variant occurs most notably in southern California where the population is entirely rufous (Van Dyke, 1926)
–	Body length ≤ 6 mm except for *Pseudaptinus nobilis*; middle antennomere ratio either much longer (L/W = 3) or shorter (L/W ≤ 1.75)	9
9	Middle antennomeres markedly prolonged with L/W = 3; eye size and convexity both markedly reduced; eye diameter distinctly shorter than temple (E/T < 1); head black, contrasts with dark red-brown dorsum; punctures on forebody coarse and quite dense; elytra stretched lengthwise, slightly oval, not strongly narrowed at shoulders; 6.5-7 mm. (TX - Live Oak Co. is new US record^1^; Mexico)	*Pseudaptinus nobilis*
–	Middle antennomeres short with L/W ≤ 1.75; eyes larger with E/T ≥ 1; head not black; body length less than 6.5 mm	10
10	Eyes appear markedly enlarged against very short temples (E/T = 3); middle antennomeres shorter (L/W = 1.25); yellow-brown; 4.8-5.1 mm. (Mexico)	*Pseudaptinus simplex*
–	Eyes relatively smaller (E/T < 2); middle antennomeres longer; body usually larger	11
11	Dorsum uniformly ferruginous (reddish); eyes appear flatter and smaller against long temples (E/T = 1); middle antennomeres L/W = 1.5; 5.1 - 5.5 mm. (western US: AZ^1, 2^, CA, OR, TX)	*Pseudaptinus rufulus*
–	Dorsum not uniformly reddish, often darker brownish; eyes larger relative to temples; middle antennomeres L/W = 1.75	12
12	Body length usually ≤ 5.5 mm; eyes relatively large with temples shorter, strongly curved (E/T = 1.5-1.9); see also key step #7	*Pseudaptinus dorsalis*
–	Body length 5.6-6 mm; eyes relatively small with temples longer, gently curved (E/T = 1-1.5); see also key step #7	*Pseudaptinus hoegei*

